# Development and Molecular Cytogenetic Characterization of Cold-Hardy Perennial Wheatgrass Adapted to Northeastern China

**DOI:** 10.3389/fpls.2020.00582

**Published:** 2020-05-14

**Authors:** Wei Yan, Xin Jin, Bo Jiang, Xiaoyue Qi, Yaxin Chen, Xinling Li, Xiaoqiang Liu, Yongkang Ren, Lei Cui, Qingjie Song, Hongjie Li, Bernd Friebe, Jilin Li, Yanming Zhang

**Affiliations:** ^1^Key Laboratory of Molecular Cytogenetics and Genetic Breeding of Heilongjiang Province, College of Life Science and Technology, Harbin Normal University, Harbin, China; ^2^Institute of Crop Science, Shanxi Academy of Agriculture Sciences, Taiyuan, China; ^3^Crop Resources Institute, Heilongjiang Academy of Agriculture Sciences, Harbin, China; ^4^Institute of Crop Sciences, Chinese Academy of Agricultural Sciences, Beijing, China; ^5^Department of Plant Pathology, Wheat Genetics Resource Center, Throckmorton Plant Sciences Center, Kansas State University, Manhattan, KS, United States

**Keywords:** perennial wheatgrass, *Thinopyrum intermedium*, cold hardiness, molecular cytogenetic, chromosome

## Abstract

Cold-hardy perennial wheatgrass plays an important role in the use of barren land for farming, soil and water conservation, variety improvement, and also for increasing grass yield. By crossing octoploid tritelytrigia (2n = 8x = 56, AABBDDEE) with *Thinopyrum intermedium* (2n = 6x = 42, StStJJJ^*S*^J^*S*^), we developed 34 lines of perennial wheatgrass from F_1_ to F_6_ generations, which had vigorous regrowth and cold hardiness. The cold-hardy, perennial wheatgrass lines were well-adapted to the cold environment and developed root and rhizomes, with a longevity between 5 and 11 years and a better seed set. Some of them maintained wheat chromosomes beneficial for breeding perennial wheat. Molecular cytogenetic analysis demonstrated that the *Th. intermedium* chromosomes contributed the most to the synthetic genome of the wheatgrass hybrids and were associated with the perennial growth habit and winter hardiness. They were also preferentially maintained and transmitted to the progenies. Some wheat chromosomes were also transmitted from the F_1_ to F_6_ generations, although they were eliminated in each life cycle of the wheatgrass hybrids. The numbers of wheat and *Th. intermedium* chromosomes affected seed set and perennial growth habit. Seed set increased with the establishment of a more balanced genomic constitution in later generations. The cold-hardy and perennial wheatgrass lines were produced, which can be the starting point of domestication effort aimed at producing well-adapted ground cover plants under extreme environments.

## Introduction

Security in food and ecosystems is benefited from perennial grain crops that provide additional options for diverse and marginal conditions (Glover et al., 2007, 2010). Perennial grain crops offer a continuous soil cover that prevents soil erosion, improves nutrient balance against soil acidification, increases water tables and salinity and ultimately benefit improvement of ecosystems (Larkin et al., 2014). Many related species of common hexaploid wheat (*Triticum aestivum* L.) are perennial, for example, wheatgrasses *Thinopyrum intermedium* (Host) Barkworth & D. R. Dewey (2n = 6x = 42, StStJJJ^*S*^J^*S*^) and *Th. ponticum* (Podp.) Barkworth D. R. Dewey (2n = 10x = 70, J^*S*^J^*S*^J^*S*^J^*S*^JJJJJJ). However, they cannot be directly grown as perennial grain crops.

The dream of developing perennial wheat can be dated back to the works of Dr. N. V. Tsitsin in the 1920s to the 1930s (Tsitsin, 1978). Two strategies have been applied to develop perennial grain crops, domestication of wild species and hybridization between cultivated crops with their perennial relatives (Cox et al., 2002). One of the successful examples is the release of Montana-2 (MT-2) in Montana, United States (Schulz-Schaeffer and Haller, 1987). This perennial wheat was developed by crossing durum wheat (*Triticum turgidum* L. var. *durum*) and *Th. intermedium* at Montana State University, Bozeman, MT, United States. Cytological analysis indicates that this line had 32 wheatgrass chromosomes in addition to about 24 wheat chromosomes (Schulz-Schaeffer and Friebe, 1992).

Recently, efforts to develop perennial wheat have been independently initiated by The Land Institute (Cox et al., 2010) and Washington State University in the United States (Murphy et al., 2010). The hybridization between the perennial wild wheat relatives and common wheat produced 610 perennial lines with different number of alien chromosomes (Lammer et al., 2004; Cox et al., 2006). Murphy et al. (2007) analyzed four pasture-type, wild wheat relatives and used them as the parents to develop a perennial wheat suitable for drier regions of the U.S. Pacific Northwest. Bell et al. (2008, 2010) reviewed the challenges in developing perennial wheat that could improve grain production systems in Australia by rectifying many environmental problems. Among 176 wheat-wheatgrass lines, 107 showed some ability to regrow after the sexual cycles over three consecutive years of cultivation in two Australian locations (Hayes et al., 2012). A network of 21 experiments was established in nine countries on the four continents across both hemispheres to evaluate the performance of early generation perennial materials derived from wheat, rye (*Secale cereal* L.) and barley (*Hordeum vulgare* L.) (Hayes et al., 2018). However, only a few perennial wheat lines were able to grow continuously under natural condition for more than 3 years, especially in cold regions.

The dispute in chromosome compositions of perennial wheat has never stopped. Variation in the genomic constitutions of the perennial wheat lines impacts plant performances, such as fertility, regrowth ability, cold hardiness, and perenniality. Lammer et al. (2004) examined single-chromosome addition and substitution lines of *Th. elongatum* to Chinese Spring wheat and concluded that chromosome 4E of *Th. elongatum* conferred a capacity for post-harvest regrowth (PHR) in wheat. It seems that at least one gene is responsible for the regrowth trait on chromosome 4ES; however, the ability to regrow and polycarpy of the disomic CS-*Th. elongatum* addition line 4E (CS + 4E”) was not comparable as the perennial amphiploid line carrying the complete set of *Th. elongatum* chromosomes. It is likely that many genes on different alien chromosomes are required to confer full perennial growth habit (Zhao et al., 2012). Cox et al. (2006) proposed that more than one wheatgrass genome was required for perennial wheat to live in the field for many years. Hayes et al. (2012) conducted that if the wheat parent was hexaploid, the progeny lines required at least 56 chromosomes to achieve substantial PHR. Nevertheless, the presence of 56 chromosomes is not a guarantee of a capability to survive post-harvest.

Larkin et al. (2014) concluded that the best near-term prospect for a perennial, wheat-like grain crop is a full or partial amphiploid containing a full set of tetraploid (AABB) or hexaploid (AABBDD) wheat chromosomes plus one genome equivalent (XX) from the donor species of perenniality. To learn what percentage of wheatgrass and wheat chromosomes is appropriate for stronger perenniality, the Land Institute of the United States developed a panel of full amphiploids with different genomic constitutions that were composed of different ratios of wheat-wheatgrass chromosomes (DeHaan et al., 2013). However, no perennial lines with wheat genomes has been reported to survive in the cold regions for more than 3 years.

In China, hybridization between wheat and wheatgrasses was initiated in the 1950s. In 1953, hexaploid wheat and *Th. intermedium* were crossed resulted in the production of a series of partial amphiploids, e.g., the “Zhong” series lines (Sun, 1981). Such crosses have been used to develop perennial wheat in Shanxi province, China (Sun et al., 2011; Cui et al., 2018). *Th. intermedium* was also crossed with the octoploid tritielytrigia lines (2n = 8x = 56, AABBDDEE) that were developed from the cross between common wheat line 91C-9 and the partial amphiploid line Yuan 16-3 for developing cold-hardy perennial wheat and wheatgrass in Heilongjiang province in northeastern China, where has a harsh cold winter climate (Yan et al., 2010; Zhao et al., 2012; Jin et al., 2014). In this area, perennial wheat must have cold-hardiness to ensure the perennial performance. Since 2006, we have been working on developing perennial wheat and wheatgrass by crossing octoploid tritelytrigia (2n = 8x = 56, AABBDDEE) with perennial *Th. intermedium*. We obtained new perennial lines with excellent cold-hardiness. Here, we report the development and cytogenetic characterization of novel cold-hardy perennial wheat-wheatgrass lines.

## Materials and Methods

### Plant Materials

The annual octoploid partial amphiploids Ganmai 8 and Ganmai 9 (2n = 8x = 56, AABBDDEE) are sister lines that were developed from the cross between common wheat line 91C-9 and the partial amphiploid line Yuan 16-3 in Shanxi Academy of Agricultural Sciences, Taiyuan, Shanxi province. The cold-hardy *Th. intermedium* (2n = 6x = 42, StStJJJ^*S*^J^*S*^) accession originated from Russia and is currently maintained in Heilongjiang Academy of Agricultural Sciences, Harbin, Heilongjiang province. An accession of *Pseudoroegneria strigose* (Schult. A. Löve) (2n = 14, StSt) and Chinese Spring wheat were included as the sources of DNA to be used as a probe and a blocker to detect *Th. intermedium* chromosomes. *Roegneria ciliaris* (Trin.) Nevsk (2n = 28, StStYY) (Trin.) Nevsk was used as control in molecular test.

### Development of Perennial Wheat Lines and Cold-Hardiness Tests

Ganmai 8 and Ganmai 9 as the annual maternal parents were crossed with *Th. intermedium*. The F_1_ plants were perennial but sterile in the first year of growth. A few seeds were obtained from the second year. Similar performance was observed from F_2_ to F_6_ generations. Seeds obtained from the first harvest of each generation were germinated at 23°C and the plants were transplanted into the fields in April in 2-m rows and 0.2-m spacing between plants. Adjacent rows were spaced 0.3 m apart. From the F_5_ to F_6_, 30 seeds were sown directly into the field for examining seed germination.

Plots for testing cold-hardiness were established in a farm at Harbin Normal University (126°57′ E, 45°87′ N), Harbin, China. The agricultural climate of Harbin is warm semiarid, with spring drought and a semi-humid summer. The annual average temperature is 3.3°C, −19.4°C in January and 23.1°C in July. The extreme winter temperature is −35°C. Plants were allowed selfing in the fields, seed harvest usually occurred in August, but varied depending upon maturity of individual plants. Grains were harvested in autumn and the above-ground parts were cut at 10 cm above the soil surface. The PHR was visually evaluated from harvest to the onset of winter in October and November. The regrowth of underground rhizomes was also examined. From November to early April in the following year, plants in the field were exposed to the natural cold environment to examine their cold hardiness. If plants could survive during the winter and regrow for at least three consecutive years, they were regarded as cold-hardy perennials. Ganmai 8, Ganmai 9, and the *Th. intermedium* accession were grown each year in 2-m rows and 0.2-m spacing as the annual and perennial controls, respectively. Morphological observations were as described previously (Li and Li, 2006). Before harvest, about 20 plants of each line were randomly selected for trait investigation, including plant height, tiller number, spike length, spikelet number per spike, underground rhizome, cold hardiness, and growth period.

### Cytogenetic Analysis

Cytogenetic analyses for the perennial wheat entries included chromosome counting of the root tip cells, meiotic behaviors of chromosomes, and characterization of chromosome constitutions by genomic *in situ* hybridization (GISH) and Oligonucleotide *in situ* hybridization. Seeds were germinated at 23°C for 24 h, 4°C for 48 h, and 23°C for 27.5 h. Root tips were incubated in ice water at 0–4°C for 24 h and fixed in Carnoy’s solution (ethanol: glacial acetic acid = 3:1) for 24 h. They were stained in 1% aceto-carmine for at least 5 h prior to squashing in 45% acetic acid. Chromosome numbers were counted under a light microscope (Leica DM LS2, Mannheim, Germany). Inflorescences were sampled at 8:00 to 9:00 am or 15:00 to 16:00 pm, fixed in Carnoy’s solution for 5–10 h, stored in 75% ethanol, and anthers at appropriate stages were stained in 1% aceto-carmine. Chromosome behaviors were observed under a light microscope.

DNA extraction from fresh leaves was performed using the CTAB method (Doyle and Doyle, 1987). The St-genome DNA as probe can be used for GISH analysis of *Th. intermedium* chromosome (Chen et al., 1998; Chen, 2005). The probe was prepared by labeling 1 μg (2 μl) *Ps. strigose* and common wheat “Chinese Spring” genomic DNA in 4 μl of DIG-Nick-Translation mix (Roche, Mannheim, Germany) and 14 μL of ddH_2_O at 15°C for 90 min. The reaction was terminated by 1 μL 0.5 mol EDTA (pH 8.0) at 65°C for 10 min. DNA of Chinese Spring wheat and *Th. intermedium* were sheared to be used as a blocker. Anti-digoxin Rhodamine and DAPI (Roche, Mannheim, Germany) were added prior to incubation in the dark. A Leica DM6000B fluorescence microscope (Leica, Mannheim, Germany) was used for observing the hybridization signals, and images were captured with a Leica digital camera (Model DFC480).

An oligonucleotide (oligo hereafter) multiplex containing oligos *pAs1-1*, *pAs1-3*, *AFA-4 (GAA) 10*, and *pSc119.2-1* was used in oligonucleotide *in situ* hybridization to discriminate wheat chromosomes. The synthetic oligo *pAs1-1*, *pAs1-3*, and *AFA-4* were 5′ end-labeled with 6-carboxytetramethyl-rhodamine (TAMRA) for red signals. The synthetic oligo *pSc119.2-1* and *(GAA) 10* were 5′ end-labeled with 6-carboxyfluorescein (6-FAM) for yellow-green signals. Genomic DNA from *Ps. strigose* was labeled with fluorescein-12-dUTP by the nick translation method as described above and used as a probe for bright green signals. The protocol of GISH/FISH using the synthesized probes was previously described by Wang et al. (2017).

### Molecular Marker Analysis

Universal E-genome primers P3 and P4 (5′-GCTGAATCTGCGTATCGTCCC-3′ and 5′-GACTTGTTCTTCGGCGTGTTG-3′), and St-genome primers St_542_ (5′-CTTCCGCAGTCCTGTGTGAAG-3′) and (5′-TAGATGATGTCTCACGCTCAC-3′) were described by You et al. (2002) and Liu et al. (2007) The reaction mixture (25 μL) contained 15.7 μL deionized water, 2.5 μL of 10 × buffer, 2.5 μL of dNTP, 1 μL of primer R and primer L each, 0.3 μL of RT-*Taq*, and 2 μL (10–30 ng) of DNA. Amplification conditions were 94°C for 5 min and 40 cycles of 94°C for 30 s, 37°C for 45 s, and 72°C for 1 min, followed by 72°C for 10 min. An Eppendorf 9700 thermocycler (Eppendorf, Hamburg, Germany) was used for DNA amplification, and PCR products were examined by agarose gel electrophoresis.

## Results

### Development of Cold-Hardy Perennial Hybrids

**FIGURE 1:**
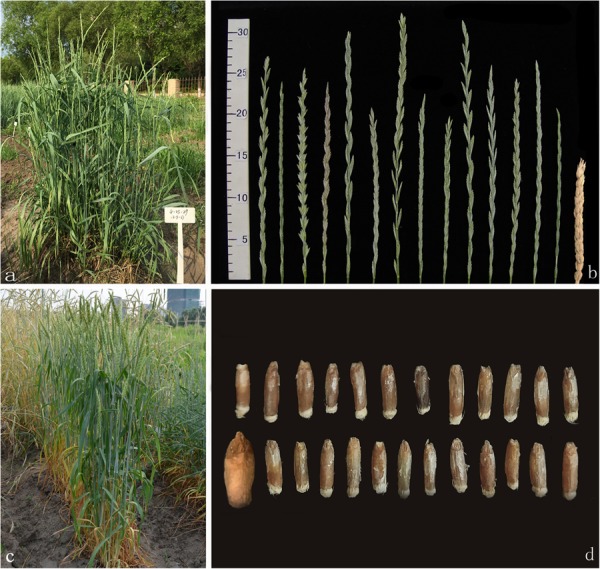
Plant, spikes and seeds of the perennial wheatgrasses and parents. **(a)** The plant of F_6_ line 42529356 in the field (photo taken in June 2016). **(b)** Spikes of the wheatgrass plants from F_1_ to F_4_ generations and parents. The first-left spike is *Th*. *intermedium* and the first-right spike is Ganmai 8. **(c)** The plant of Ganmai 8 in the field (photo taken in June 2016). **(d)** Seed characteristics of perennial wheatgrasses and parent. A seed of Ganmai 8 and *Th. intermedium* are shown on the left of the picture. The top-left seed is *Th. intermedium* and the bottom-left seed is Ganmai 8.

Since 2007, F_1_–F_6_ hybrids between the two octoploid partial amphiploids, Ganmai 8 and Ganmai 9, and *Th. intermedium* were grown under the natural cold winter conditions in Harbin. Out of 1200 lines, 34 survived and showed perennial growth habit (Table 1). They were regarded as the wheatgrass type because they resembled their paternal parent *Th. intermedium* (Figures 1a,c).

**TABLE 1:** Cold-hardy test of the wheatgrass hybrids from 2007 to 2018 generations.

**Generation**	**No. of lines**	**Years of growth**
F_1_	9	9–11
F_2_	4	8–10
F_3_	7	8
F_4_	2	7
F_5_	4	6
F_6_	8	5

Twenty F_1_–F_3_ lines have grew for 8–11 years under the local conditions. Nine F_1_ lines, 1-11-1, 1-11-2, 1-11-3, 1-11-4, 1-12-1, 1-12-2, 1-12-3, 1-12-4, and 1-12-5, four F_2_ lines, 1-1-4, 2-19-1, 1-2-10, and 1-6-2, and seven F_3_ lines, 1-18-1, 1-15-1, 1-15-2, 1-15-3, 1-15-4, 1-7-4, and 2-11-1, were perennial. The F_1_ plants were sterile in the first growth year, because they were heptaploids with poor chromosome pairing during meiosis. However, these plants were able to regrow and survive during the winter. After the second growing year, a few seeds were produced in these plants. Five years later in the field, the chromosome count of the F_1_ lines reduced to 2n = 42. The seed set of these plants increased, and the best line, 1-11-3, had a percentage of seed set up to 44% (Table 2), but the seeds were smaller than wheat.

**TABLE 2:** Morphological traits of nine perennial wheatgrass lines from F_1_ hybrids in 2013.

**Trait**	***Th. intermedium***	**Ganmai 8**	**1-11-1**	**1-11-2**	**1-11-3**	**1-11-4**	**1-12-1**	**1-12-2**	**1-12-3**	**1-12-4**	**1-12-5**
Plant height (cm)	104 ± 1.3	97 ± 2.1	85 ± 1.4	94 ± 1.1	93 ± 2.4	118 ± 1.4	123 ± 1.7	113 ± 1.4	105 ± 2.3	107 ± 1.9	102 ± 2.3
No. of tillers	168 ± 2.2	6 ± 1.2	2 ± 1.5	29 ± 1.6	24 ± 2.7	53 ± 1.7	34 ± 1.4	46 ± 0.4	51 ± 2.1	46 ± 1.5	24 ± 1.8
Spike length (cm)	17.8 ± 1.4	13.8 ± 1.3	9.8 ± 1.4	17.6 ± 1.3	15.8 ± 1.6	18.2 ± 1.4	17.9 ± 1.4	17.5 ± 1.5	23.6 ± 1.2	17.3 ± 1.5	16.9 ± 1.2
No. of spikelets	18 ± 0.8	18 ± 1.4	18 ± 1.2	21 ± 1.3	23 ± 1.3	18 ± 0.6	24 ± 1.5	22 ± 0.3	19 ± 0.4	20 ± 1.3	17 ± 0.9
No. of florets	75 ± 1.4	42 ± 0.8	72 ± 1.2	84 ± 2.1	92 ± 1.1	72 ± 1.2	96 ± 1.3	88 ± 1.1	72 ± 1.0	80 ± 1.7	68 ± 1.8
Seed-setting (%)	26.3	73.3	5.1	17.4	44.2	5.2	33.3	29.9	12.2	1.0	34.3
Regrowth habit	+	−	+	+	+	+	+	+	+	+	+
Years of growth	10	−	6	6	6	6	6	6	6	6	6
Underground rhizome	+	−	+	+	+	+	+	+	+	+	+

*All indices are described by mean ± standard error; + and − indicate the presence and absence of the traits, respectively.*

In the first growth year, the F_2_ and F_3_ plants usually had chromosome numbers ranging from 50 to 56 and were poor in seed setting. After growing in the field for more than 5 years, the chromosome number of all lines reduced from 49 to 42 and the seed set percentage increased to > 80% (Table 3), which was higher than that of the male parent *Th. intermedium* (∼30%). The highest number of tillers (512) was observed in an F_3_ line 1-15-4, and this line had a mean seed set of 93.23% with the similar seed type of wheatgrass. More than three-quarters of the 150 F_1_-F_4_ plants did not resume growth in spring of 2012 partially because of no snow covering during the winter. Only two F_4_ lines, 4-31 and 5-14, survived, and the chromosome numbers and seed set were similar to that of the F_1_-F_3_ perennial hybrids in the same first growth year. Five years later in the field, the seed set percentages of these lines were > 70% (Table 3).

**TABLE 3:** Agronomic traits of 16 perennial wheatgrass lines and parents in 2017.

Line	Planting time	Years of growth	Cold hardiness	Regrowth habit	Underground rhizome	Plant height (cm)	Spike length (cm)	No. of tillers	Seed-setting (%)
*Th. intermedium*	2003	14	+	+	+	106.50 ± 1.3	16.8	270	28.3
Ganmai 8	2017	1	−	−	−	96.8 ± 1.4	14.5	7	76.2
F_2_ 1-1-4	2009	8	+	+	+	118.30 ± 2.6	16.57	80	81.41
F_2_ 2-19-1	2009	8	+	+	+	107.34 ± 1.5	16.23	310	88.52
F_3_ 1-18-1	2010	7	+	+	+	105.00 ± 1.7	18.20	312	94.21
F_3_ 1-15-4	2010	7	+	+	+	122.45 ± 2.1	20.12	512	93.24
F_4_ 5-14	2011	6	+	+	+	122.10 ± 1.5	16.40	246	77.52
F_4_ 4-31	2011	6	+	+	+	124.31 ± 1.3	15.21	342	80.53
F_5_ 4-12-13	2012	5	+	+	+	112.31 ± 2.3	19.31	430	95.24
F_5_ 4-14-15	2012	5	+	+	+	121.10 ± 3.2	18.56	323	92.55
F_5_ 4-0-8	2012	5	+	+	+	112.10 ± 2.1	17.42	125	90.36
F_5_ 4-25-29	2012	5	+	+	+	132.12 ± 1.1	16.72	211	91.42
F_6_ 42529344	2013	4	+	+	+	117.10 ± 1.5	15.31	45	23.73
F_6_ 42529912	2013	4	+	+	+	125.30 ± 1.3	22.03	315	98.32
F_6_ 41213355	2013	4	+	+	+	132.21 ± 0.9	19.57	73	34.44
F_6_ 412137111	2013	4	+	+	+	110.50 ± 1.5	17.13	62	8.93
F_6_ 412133204	2013	4	+	+	+	128.22 ± 1.6	19.17	50	83.12
F_6_ 414156	2013	4	+	+	+	155.45 ± 1.4	15.40	117	2.11

*All indices are described by mean ± standard error; + and − indicate the presence and absence of the traits, respectively.*

From 2012 and 2016 90 F_5_ hybrids were evaluated for cold hardiness and perennial growth habit. Only four lines, 4-0-8, 4-12-13, 4-14-15, and 4-25-29, survived for more than 3 years and were perennial. These hybrids had more than 2n = 50 chromosomes and low seed set for the first 3 years. The chromosome number was reduced to 2n = 42 with normal meiotic behaviors after grown in the field for 5 years and the seed set increased to more than 90% in later generations (Table 3), which was much higher than *Th. intermedium*.

**FIGURE 2:**
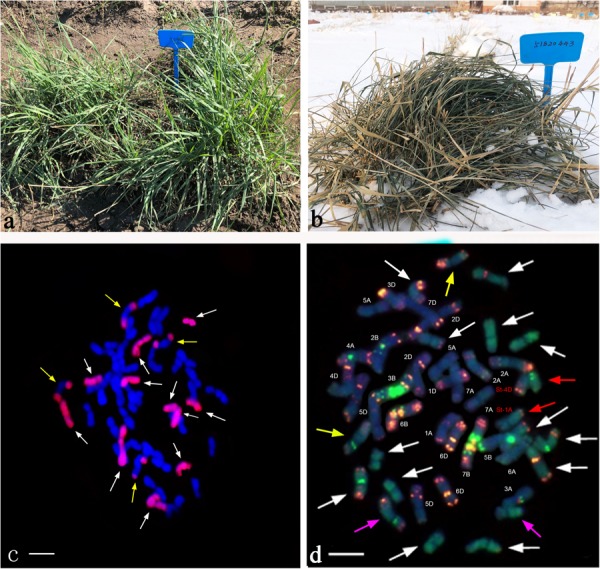
Morphological traits and GISH/FISH patterns of the perennial wheatgrasses lines. **(a)** Post-harvest regrowth (PHR) of line 42529356 (photo taken in September 2018). **(b)** Line 51820443 was covered with snow (photo taken in January 2018). **(c)** GISH pattern of line 42529356 (white arrows point to St chromosomes and yellow arrows point to J chromosomes). **(d)** GISH/FISH result of line 51820443 (white, yellow, and purple arrows point to St, J, J^*s*^ chromosomes, respectively. Red arrows point to St-wheat translocated chromosome).

During 2013 to 2019, eight F_6_ lines survived the cold winter and showed perennial growth habit. Six of them, 42529344, 41213355, 412137111, 42529912, 414156, and 412133204, had 2n = 42 chromosomes. Line 42529912 and 412133204 kept good seed set from the first life cycle to present that was more than 80% in 2017 (Table 3). Meanwhile, the tiller number of line 42529912 (315) was highest in the F_6_. The other two lines, 42529356 and 51820443 (Figures 2a,b), were sterile in the first 2 years and seed set was 36.13 and 1.28%, respectively (Table 4). The chromosome number of these lines ranged from 2n = 45 to 50. The tiller number of line 42529356 increased from 81 to 296, but decreased from 118 to 36 for line 51820443 over a 3-year period. These lines were subjected to full characterization in morphological, cytological, and molecular levels. They were the first materials in the F_6_ generation to have the cold-hardy perennial trait and keeping more than 2n = 42 chromosomes.

**TABLE 4:** Agronomic traits of two F_6_ perennial wheatgrass lines during 2016 and 2018.

**Line**	**Years of growth**	**Underground rhizome**	**Plant height (cm)**			**Spike length (cm)**			**No. of tillers**			**Seed-setting (%)**		
			
			**2018**	**2017**	**2016**	**2018**	**2017**	**2016**	**2018**	**2017**	**2016**	**2018**	**2017**	**2016**
42529356	5	+	105	133	147	16.2	17.23	23.7	296	275	81	17.33	18.07	36.13
51820443	5	+	92	87	110	16.5	14.33	15.8	34	70	118	0	3.92	1.28

*+ and − indicate the presence and absence of the traits, respectively.*

### Morphological Performance of the Hybrids in the Field

Spikes of the F_1_ and F_2_ hybrids were classified as wheatgrass type based on their morphologies of spikes (Figure 1b). The common wheat type spikes appeared in the F_3_ plants, which had 2n = 42 chromosomes. The wheatgrass-type plants were perennial, but the wheat-type plants were annual. In the F_4_ and F_5_ generations, a third spike type, designated the tritelytrigia type, was observed. However, most of them were annual and only a few grew in the field for 2–3 years. The regrowth hybrids only produced a few seeds with poor agronomic performances. The chromosome numbers of these lines varied from 2n = 48 to 56. The annual plants resembled the octoploid tritelytrigia parents, Ganmai 8 or Ganmai 9. In the F_6_ generation, only the wheatgrass-type plants survived and had perennial growth habit.

**FIGURE 3:**
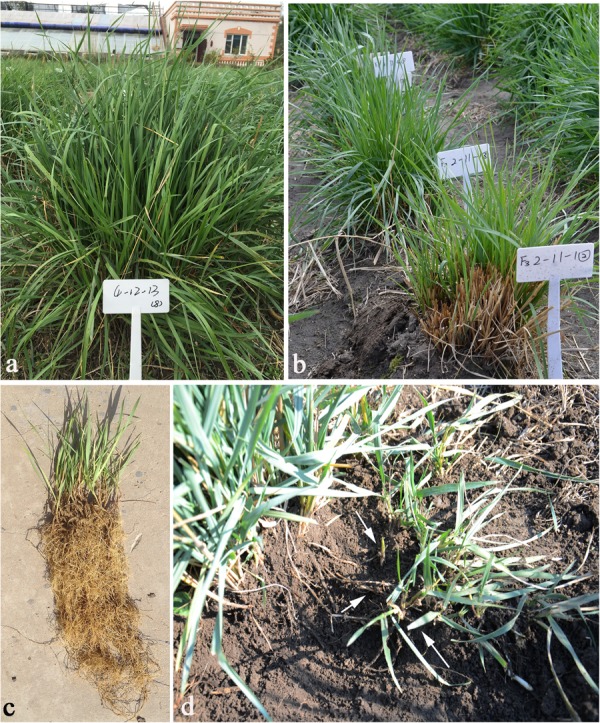
Morphological traits of the perennial wheatgrasses in the field. **(a)** PHR of F_5_ 4-12-13 in September, 2015. **(b)** Plants of F_3_ 2-11-1 resumed to vegetative growth state after winter in May, 2014. **(c)** Developing roots of F_3_ 1-18-1 in the spring. **(d)** Regrowth of F_3_ 1-18-1 from underground rhizomes during September and November, 2013. Arrows show the underground rhizomes and regrowth plant.

From the F_1_ to F_6_ generations, 34 lines of the wheatgrass-type plants were lush, with an average plant height > 1 m, and except F_6_ line 51820443, the number of tillers increased with the number of regrowth seasons (Tables 2–4). All the hybrid plants had PHR after August and the regrowing plants were able to grow up to the end of November (Figure 3a). During December and January, the leaves and stems of such plants remained green under the snow-cover. In March and April, the leaves and stems were withered. After cutting, new leaves regrew from the original crowns and the plants returned to the vegetative state for a new life cycle in May (Figure 3b). The perennial hybrid plants had well-developed roots and almost underground rhizomes after living for more than 3 years (Figures 3c,d). They matured at August, 1 month earlier than the first growth year. Grains obtained from these perennial plants became full and smooth with the increase of life cycles. Some grains, either red or blue in color, were larger than those of the *Th. intermedium*, but smaller than Ganmai 8 and Ganmai 9 (Figure 1d). The seeds did not germinate in the field during the first 5 years, but a few germinated thereafter.

### Molecular Analysis With the Genome-Specific Primers P3, P4, and St_542_

**FIGURE 4:**
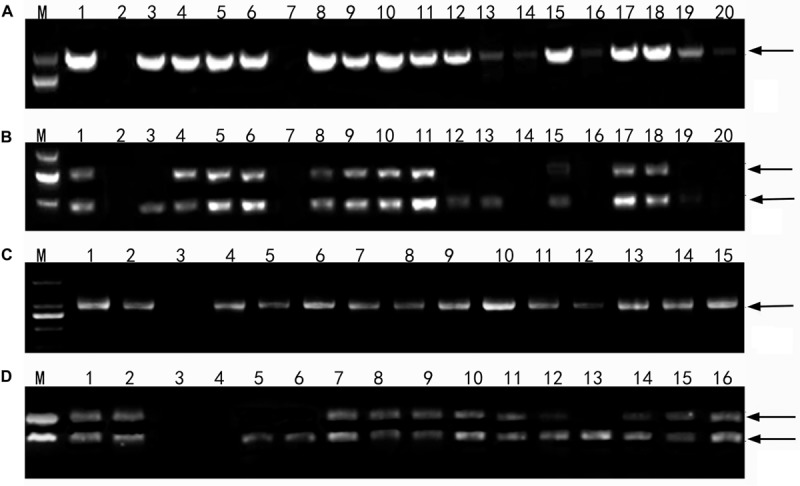
Profiles of DNA amplification of the wheatgrass progeny lines with the genome specific molecular markers. **(A)** An amplification profile of F_1_-F_4_ hybrid plants using the primers P3 and P4 specific for the E genome. Marker: DNA ladder DL2000. Lane 1 *Th. intermedium*, lane 2 Chinese Spring, lane 3 Ganmai 8, lane 4 Ganmai 9, lane 5 F_1_ 1-11-3, lane 6 F_1_ 1-11-4, lane 7 F_1_ 1-12-1, lane 8 F_1_ 1-12-2, lane 9 F_1_ 1-12-3, lane 10 F_1_ 1-12-4, lane 11 F_1_ 1-12-5, lane 12 F_2_ 2-19-1, lane 13 F_2_ 1-1-4, lane 14 F_3_ 1-18-1, lane 15 F_3_ 1-15-1 lane 16 F_3_ 1-15-2, lane 17 F_3_ 1-15-3, lane 18 F_3_ 1-15-4, lane 19 F_4_ 5-14, and lane 20 F_4_ 4-31. **(B)** An amplification profile of F_1_-F_4_ hybrid plants using primers *St*_542_ specific for the St genome. Marker: DNA ladder DL2000. Lane 1 *Th. intermedium*, lane 2 Chinese Spring, lane 3 *Ps. strigosa*, lane 4 *R. ciliaris*, lane 5 F_1_ 1-11-3, lane 6 F_1_ 11-4, lane 7 F_1_ 1-12-1, lane 8 F_1_ 1-12-2, lane 9 F_1_ 1-12-3, lane 10 F_1_ 1-12-4, lane 11 F_1_ 1-12-5, lane 12 F_2_ 2-19-1, lane 13 F_2_ 1-1-4, lane 14 F_3_ 1-18-1, lane 15 F_3_ 1-15-1, lane 16 F_3_ 1-15-2, lane 17 F_3_ 1-15-3, lane 18 F_3_ 1-15-4, lane 19 F_4_ 5-14, and lane 20 F_4_ 4-31. **(C)** An amplification profile of F_5_ and F_6_ perennial hybrid plants using primer P3 and P4. Marker: DNA ladder DL2000. Lane 1 *Th. intermedium*, lane 2 *Th. elongatum*, lane 3 Chinese Spring, lane 4 F_6_ 42529356, lane 5 F_6_ 51820443, lane 6 F_6_ 42529344, lane 7 F_5_ 4-0-8, lane 8 F_5_ 4-14-15, lane 9 F_5_ 4-12-13, lane 10 F_5_ 4-25-29, lane 11 F_6_ 412137111, lane 12 F_6_ 41213355, lane 13 F_6_ 42529912, lane 14 F_6_ 414156, lane 15 F_6_ 412133204. **(D)** An amplification profile of F_5_ and F_6_ hybrid plant using primer *St*_542_. Marker: DNA ladder DL2000. Lane 1 *Th. intermedium*, lane 2 *P. strigose*, lane 3 *Th. elongatum*, lane 4 Chinese Spring, lane 5 F_6_ 42529356, lane 6 F_6_ 51820443, lane 7 F_6_ 42529344, lane 8 F_5_ 4-0-8, lane 9 F_5_ 4-14-15, lane 10 F_5_ 4-12-13, lane 11 F_5_ 4-25-29, lane 12 F_6_ 412137111, lane 13 F_6_ 41213355, lane 14 F_6_ 42529912, lane 15 F_6_ 414156, and lane F_6_ 412133204. Arrows indicate the target amplification products.

Sixteen lines of the perennial wheatgrass hybrids from the F_1_-F_4_ generations were characterized using the molecular markers specific for the E and St genomes. The E genome-specific primers P3 and P4 produced a 982 bp band in 15 lines, except for line 1-12-1 and Chinese Spring (Figure 4a), indicating that they contained the E-genome chromatins. The St-genome-specific primer St_542_ amplified the expected 750- and 450-bp bands in 12 progeny lines, and *Th. intermedium*, *P. strigose*, indicating the presence of the St-genome chromatins in these materials (Figure 4b and Table 5). Four lines, F_1_ 1-12-1, F_3_ 1-18-1, F_3_ 1-15-2, and F_4_ 4-31, failed to produce the expected bands, indicating the absence of the St-genome chromatins. During the 2016 and 2017 cropping seasons, 8 F_6_ and 4 F_5_ perennial lines were genotyped with the E- and St-genome specific molecular markers. All the lines examined produced the 982-bp product with P3 and P4 markers (Figure 4c) and the 750-bp and 450-bp bands with the St_542_ marker (Figure 4d and Table 5). These results indicate that the perennial hybrids from the F_5_ and F_6_ generation had retained the genomic components of *Th. intermedium*.

**TABLE 5:** Analysis molecular markers of the perennial wheatgrass hybrids from F_1_ and F_6_ generations.

**Line**	**No. of chromosomes**	**P3-P4**	**St_542_**	**Line**	**No. of chromosomes**	**P3-P4**	**St_542_**
F_1_ 1-11-3	42	+	+	F_4_ 5-14	42	+	+
F_1_ 1-11-4	42	+	+	F_4_ 4-31	42	+	−
F_1_ 1-12-1	42	−	−	F_5_ 4-0-8	42	+	+
F_1_ 1-12-2	42	+	+	F_5_ 4-14-15	42	+	+
F_1_ 1-12-3	42	+	+	F_5_ 4-12-13	42	+	+
F_1_ 1-12-4	42	+	+	F_5_ 4-25-29	42	+	+
F_1_ 1-12-5	42	+	+	F_6_ 42529356	45–50	+	+
F_2_ 2-19-1	42	+	+	F_6_ 51820443	45–50	+	+
F_2_ 1-1-4	42	+	+	F_6_ 42529344	42	+	+
F_3_ 1-18-1	42	+	−	F_6_ 42529912	42	+	+
F_3_ 1-15-1	42	+	+	F_6_ 41213355	42	+	+
F_3_ 1-15-2	42	+	−	F_6_ 412137111	42	+	+
F_3_ 1-15-3	42	+	+	F_6_ 412133204	42	+	+
F_3_ 1-15-4	42	+	+	F_6_ 414156	42	+	+

*+ and − indicate the presence and absence of the traits, respectively.*

### GISH Analysis of Different Types of Wheat-Wheatgrass Hybrids

**FIGURE 5:**
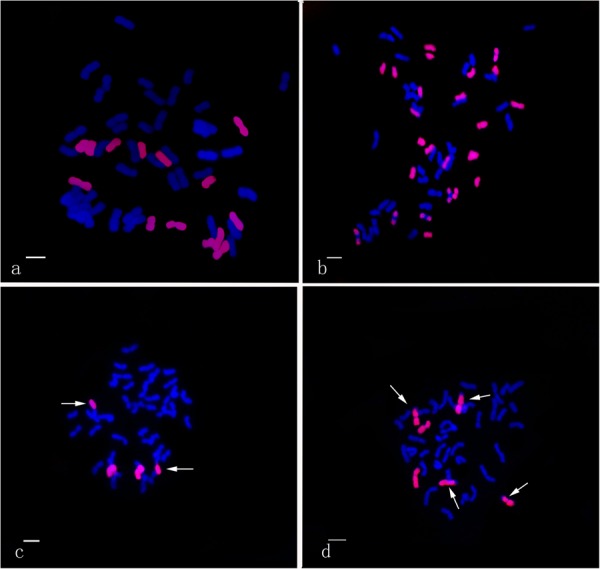
GISH patterns of the perennial wheatgrasses lines and parent. **(a)** GISH pattern of Ganmai 8 (2n = 8x = 56, 14 chromosomes were pink signals with *Th. intermedium* as probe and Chinese Spring as blocker). **(b)** GISH pattern of F_5_ 425294 (2n = 8x = 56, 24 chromosomes were pink signals with St-genome DNA as probe and Chinese Spring as blocker). **(c)** GISH pattern of line F_6_ 412133204 (2n = 6x = 42, 4 chromosomes were pink signals with common wheat “Chinese Spring” DNA as probe and *Th. intermedium* as blocker, including two wheat-wheatgrass translocated chromosomes. Arrows point to translocated chromosomes). **(d)** GISH pattern of line F_6_ 414156 (2n = 6x = 42, 6 chromosomes were pink signals with common wheat “Chinese Spring” DNA as probe and *Th. intermedium* as blocker, including four wheat-wheatgrass translocated chromosomes. Arrows point to translocated chromosomes).

Using the *Th. intermedium*-genome DNA probe, GISH analysis demonstrated that Ganmai 8 and 9 with 2n = 56 chromosomes were composed of 42 wheat chromosomes and 14 *Th. intermedium* chromosomes (Figure 5a). Using the St-genome DNA probe, examination of F_5_ plants that had only survived for 2–3 years, indicated that these plants contained 28 to 42 wheat chromosomes and 14 to 24 *Th. intermedium* chromosomes, resulting in 48 to 56 chromosomes in total. Such as line 425294, GISH analysis showed that 24 chromosomes showing hybridizing signals with St-genomic probe. 20 chromosomes belonged to the St genome with the whole signals and four belonged to the J chromosome with the terminal signals. The rest of chromosomes from wheat displayed the blue were 32 (Figure 5b).

The perennial wheatgrass type of hybrids (2n = 42 = 46) varied in their chromosome compositions. Using St-genome and common wheat “Chinese spring” DNA probe, GISH analysis demonstrated that 30 lines of wheatgrass with 2n = 42 chromosomes from F_1_ to F_6_ has no wheat chromosomes and their genomes were consisted of *Th. intermedium* chromosomes. Only four lines from F_6_, 412133204, 414156, 42529356 and 51820443, showed a difference of genome. Line 412133204 with 2n = 42 chromosomes mainly consisted of *Th. intermedium* chromosomes with 2 wheat chromosomes and 2 wheat-wheatgrass translocated chromosomes (Figure 5c). Line 414156 with 2n = 42 chromosomes consisted of *Th. intermedium* chromosomes with 2 wheat chromosomes and 4 wheat-wheatgrass translocated chromosomes (Figure 5d).

In order to determine the genome composition of the perennial hybrids, both St genome and oligo-probes were used to distinguish between wheat and *Th. intermedium* chromosomes. Based on the fluorescent signal patterns, the chromosome compositions of two lines with cold-hardiness were analyzed in detail. The F_6_ line 42529356 (2n = 46) consisted of 11 St-genome chromosomes with bright pink fluorescence hybridization signals along the entire chromosomes; 4 J-genomes chromosomes that displayed a light-blue fluorescence signals over most of their lengths except for the terminal regions. The remaining chromosomes originated from common wheat. So, this line had 15 complete chromosomes from *Th. intermedium* and 31 complete wheat chromosomes (Figure 2c). Another F_6_ line 51820443 (2n = 45) contained 13 St-genome chromosome, 2 J-genome chromosome, 2 J^*s*^-genome and 2 St-wheat genome translocated chromosome, St-4D and St-1A, and 26 wheat chromosomes. Oligo-probes showed that wheat chromosomes contained 10 A-genome chromosome (1A, 3A, 4A, 6A, and a pair of 2A, 5A, and 7A), 5 B-genome chromosome (2B, 3B, 5B, 6B, and 7B), and 11 D-genome chromosome (1D, 3D, 4D and a pair of 2D, 5D, 6D, and 7D) (Figure 2d). This indicates that wheat chromosomes can be maintained in high numbers in the new wheatgrass hybrids up to the F_6_ generation.

## Discussion

The adaptability of perennial plants to the local environment is crucial for their survival. In the cold-temperate climates, perennial plants must adapt to seasonal changes and abiotic stresses, such as frost, to be able to regrow for several years (Westerbergh et al., 2018). In the cold regions, the cold hardiness of plant is a prerequisite for ensuring perennial performance. The continuous selection under the natural winter conditions can reflect the adaptation of plants to the environment. Meanwhile, it also plays a role in the domestication of a wild species, so the wheat-wheatgrass progenies can adapt to the cold environment even in snow-free winter. In the present study, all hybrids were required to survive in the field for at least three harvests prior to testing their winter hardiness. This allows the elimination of the hybrids that were extremely unstable and vulnerable to cold conditions. Tsitsin (1978) suggested that the winter-type regrowth was desirable for stronger perenniality. The cold-hardy perennial selections of wheatgrass type hybrids described in this study support this hypothesis. All the 34 cold-tolerant lines obtained in the last decade were perennial. The long-term field growth also was beneficial for observing the growth of root and rhizomes. All the plants grown in the field for more than 3 years had well-developed root and rhizomes and vigorous PHR ability. Some of the F_2_ to F_5_ lines had numerous tillers and better seed set than the *Th. intermedium* parent, indicating that they adapted well to the local cold environment.

A similar program for the development of perennial wheatgrass was conducted in Taiyuan, Shanxi province, China, with mild winter conditions (Cui et al., 2018). Compared to the cold winter environment in the present study, the perennial hybrids selected in Taiyuan were usually octoploid partial amphiploids (2n = 8x = 56), which cannot survive the cold winter in Harbin even if they were able to regrow for more 2 years in Taiyuan. Instead, we developed cold-hardy perennial wheat-wheatgrass hybrids, which can be the starting point of domestication efforts aimed at producing well-adapted ground cover plants under the extremely cold environments in northeastern China.

The selection for wheatgrass plants in hybridization programs is critical because the parents heavily influence the final performance of the hybrids (Hayes et al., 2018). Both parents need to be tested in the target environment (Curwen-McAdams and Jones, 2017). In this study, octoploid tritelytrigia, Ganmai 8 and Ganmai 9, and *Th. intermedium* were tested in the Harbin environment for several years. We selected the octoploid tritelytrigia as the female parent because it had good seed set and regrowth ability after harvest, but they had not cold hardness ability. Although the flowering date was later than that of common wheat, the octoploid tritelytrigia could be matched with *Th. intermedium* under our field conditions. Furthermore, the octoploid tritelytrigia genome had 2n = 42 wheat chromosomes and 14 *Th. intermedium* chromosomes, which could easily recombine with wheatgrass. *Th. intermedium* is strongly adapted to the cold winter environment. At present, the *Th. intermedium* plants still exhibit vigorous regrowth even in winter without snow cover. We also tested diploid *Th. elongatum* in our field, which had regrowth ability after harvest, but could not survive the local condition of harsh winter. Therefore, developing and selecting perennial parents adapted to the local environment is fundamental to success for breeding perennial progenies. Due to the excellent cold-hardness and perenniality, *Th. intermedium* seems to be the logical choice for breeding winter-hardy hybrids (Hayes et al., 2018).

Early generations the intergeneric hybrids are subjected to genetic shuffing beyond recombination. Loss of chromosomes, homoeologous pairing, dominance effects, and gamete inviability all skew the expression of traits and complicate selection (Udall and Wendel, 2006; Bento et al., 2011). In the present study, the genomic constitution of the crosses between octoploid tritelytrigia and *Th. intermedium* showed a large variation in the F_1_ to F_6_ generations. The F_1_ plants were sterile because of meiotic irregularities, but the plants exhibited regrowth and survived during the winter, indicating the presence of *Th. intermedium* chromosomes. Seed set rate increased with the establishment of a more balanced genomic constitution in later generations.

The F_1_ and F_2_ plants resembled *Th. intermedium* and had a strong perenniality; however, their spike morphology differed from that of *Th. intermedium*. The progenies of later generations had better seed set than *Th. intermedium*, suggesting that the genome compositions of F_1_ and F_2_ plants were different from that of *Th. intermedium* despite they share the same chromosome number of 2n = 42. We observed genetic segregation for the common wheat and the tritelytrigia types in the progenies from F_3_ to F_5_ generations. The *Th. intermedium* chromosomes were eliminated to form the common wheat-type plants with 2n = 42 chromosomes, which lost the ability for regrowth.

The plants with balanced genome constitutions of wheat and *Th. intermedium* were usually the annual *tritelytrigia* type, resembling the octoploid *tritelytrigia* parent with ∼2n = 56 chromosomes and good fertility. However, the hybrids with imbalanced genomic constitutions, even if they regrow for 2–3 years in the field, such as F_5_ line 425294, had very low fertility and regrowth property disappeared. Similar results were reported by Tsitsin (1978).

The perennial habit, the trait of interest, is characterized by the ability to continue growth after harvest and set seed for multiple years. Molecular marker and GISH analyses indicated that *Th. intermedium* chromosomes always were present in each life cycle in every generation of wheatgrass-type hybrids. However, the presence of wheat chromosomes made the hybrid genome unable to reach equilibrium in a short period of time, which led to low seed set in the early hybrid generations. Under the natural selection in cold winter environments, *Th. intermedium* chromosomes associated with perennial growth habit and winter hardiness were preferentially maintained and transmitted to the progenies. Meanwhile, the wheat chromosomes were also transmitted from F_1_ to F_6_, although some of them were eliminated in each generation. The wheat chromosomes were in balance with those of *Th. intermedium* and formed a new synthetic genome (2n = 42), containing up to 2 to 6 wheat chromosomes. Others still carried the high genome dosage of wheat in perennial hybrids of F_6_ generation, such as lines 42529356 and 51820443, but their genomes were unstable and seed set was low. This indicates that the genome constitutions of these hybrids were still not fully balanced, which was confirmed by GISH/FISH results. In two lines’ genome, the number of wheat chromosome was more than that of *Th. intermedium*, but these chromosomes were not complete sub-genome. However, fluorescent *in situ* hybridization (FISH) using oligo-probes is a very useful method for identifying species chromosomes (Komuro et al., 2013; Dechyeva and Schmidt, 2016; Pei et al., 2018). In this study, we first revealed that the genome of the 51820443 line has 10 A-genome chromosome, 5 B-genome chromosome, and 11 D- genome chromosome through oligo-probes. Although it was still difficult to accurately distinguish which wheat genome was related to cold hardiness and the perennial character, it provided a basis for chromosomal tracking and identification in offspring. Therefore, lines 42529356 and 51820443 that have been cultivated for 5 years in the cold winter environment will be a great significance for breeding true perennial wheat.

## Conclusion

The cold environment factor plays an important role for development of the perennial wheat-wheatgrass, which is conducive to domestication and selection of new cold hardness wheatgrass germplasm. At the same time, it will promote the formation of new heteropolyploid genomes between wheatgrass chromosomes and wheat chromosomes. In this process, *Th. intermedium* chromosomes will dominate and wheat chromosome will be added. However, if the genome is to be balanced, it need to take longer and larger population screens.

## Data Availability Statement

All datasets generated for this study are included in the article/supplementary material.

## Author Contributions

YZ and JL conceived and designed the research project. HL, YR, LC, and QS supervised and coordinated the research. WY, XJ, and XLL performed greenhouse and field trials. WY, YC, and BJ performed the cold-hardy perennial feature and morphological observation. WY, XQL, and XQ performed cytogenetic and molecular marker analysis. WY, YZ, HL, and BF analyzed GISH and FISH data and wrote the manuscript. All authors read and approved the manuscript.

## Conflict of Interest

The authors declare that the research was conducted in the absence of any commercial or financial relationships that could be construed as a potential conflict of interest.
